# Supplementary material for: Valorization of Quercus suber L. Bark as a Source of Phytochemicals with Antimicrobial Activity against Apple Tree Diseases
Source: Plants (Basel). 2022 Dec 7;11(24):3415. doi: 10.3390/plants11243415 (PMC9785260; doi:10.3390/plants11243415)
Supplement: Supplementary file 1 [file plants-11-03415-s001.zip › plants-2077542-supplementary.pdf]

Valorization of *Quercus suber* L. bark as a source of phytochemicals with antimicrobial activity against apple tree diseases

E. Sánchez-Hernández, V. González-García, J. Casanova-Gascón, J.J. Barriuso-Vargas, J. Balduque-Gil, B. Lorenzo-Vidal, J. Martín-Gil and P. Martín-Ramos

SUPPLEMENTARY MATERIALS

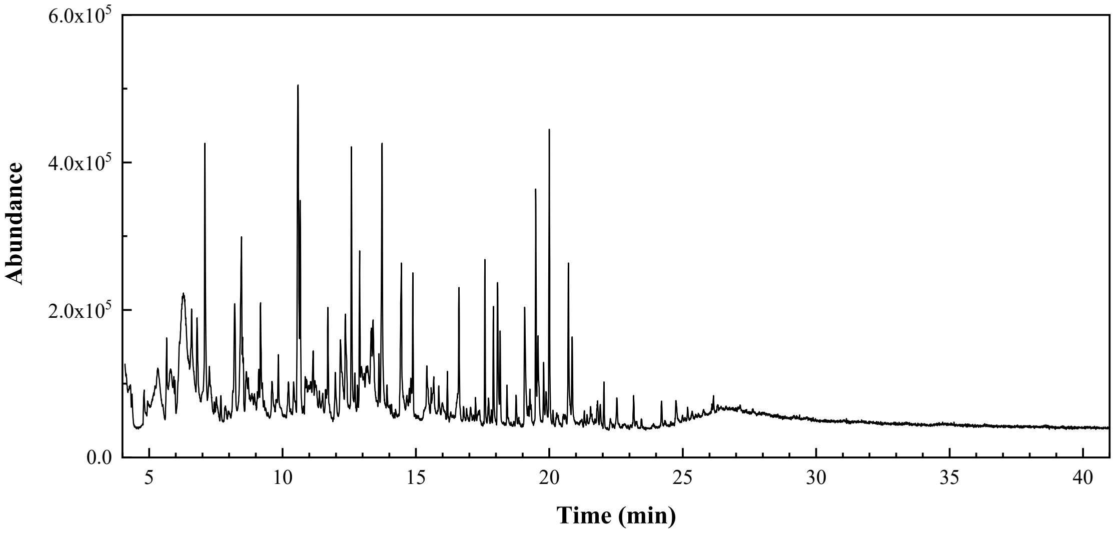

Figure S1. GC–MS chromatogram of *Q. suber* bark aqueous ammonia extract.

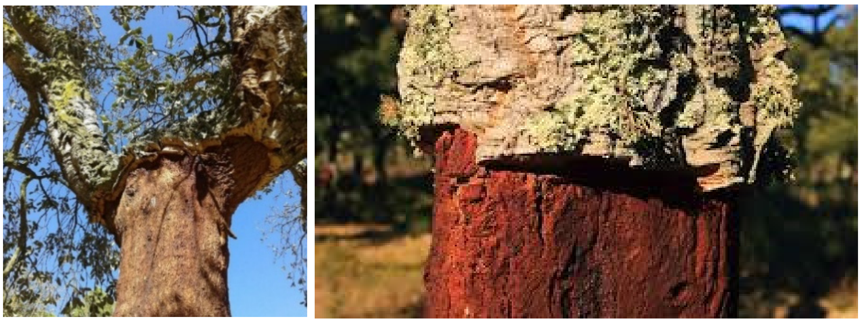

Figure S2. *Quercus suber* tree in Alcornocal de Valdegalindo, Foncastín, Valladolid, Spain

Table S1. Chemical species identified in *Q. suber* bark aqueous ammonia extract by GC−MS

Peak	RT (min)	Area (%)	Assignment
2	4.8108	0.4872	1H-Imidazole, 4,5-dihydro-2-methyl-
4	4.9474	0.5323	Diethyl carbonate
5	5.1670	0.8387	Methane, chloromethoxy-
6	5.2204	0.5830	1-Methoxy-2-methyl-3-butene
7	5.3272	2.1353	2-Furancarboxaldehyde, 5-methyl-
8	5.6596	1.0150	Hexanoic acid
12	5.9682	0.3642	3-Cyclobutene-1,2-dione, 3,4-dihydroxy-
13	6.1403	1.3106	4-Hydroxy-3-[[1,3-dihydroxy-2-propoxy]methyl]-1H-pyrazole-5-carboxamide
14	6.2887	3.8163	Glycerin
15	6.3184	3.8476	Glycerin
16	6.5974	3.0940	4-Hydroxy-3-[[1,3-dihydroxy-2-propoxy]methyl]-1H-pyrazole-5-carboxamide; 3(5)-[[1,2-Dihydroxy-3-propoxy]methyl]-4-hydroxy-1H-pyrazole-5(3)-carboxamide
17	6.7992	1.9123	α-Amino-γ-butyrolactone
18	7.0900	3.5795	2-Azabicyclo[2.2.1]heptane
27	8.4611	2.7350	Benzoic acid
28	8.5026	0.5628	Octanoic acid
29	8.5323	0.3783	α-pyrone-6-carboxylic acid
30	8.6450	0.8336	α-D-Mannopyranoside, methyl 3,6-anhydro-
33	9.1080	0.4768	Dianhydromannitol
34	9.1733	1.1565	Benzofuran, 2,3-dihydro-
36	9.6066	0.7592	Acetamide, N,N'-thiobis-
39	9.8440	0.8848	Nonanoic acid
40	10.2238	0.5838	5-Nitro-3-cyano-2(1H)-pyridone
41	10.4197	0.6269	Cycloserine
44	10.8530	0.4125	Benzamide
45	10.9005	0.4053	Benzamide
46	10.9835	0.5358	α-D-Xylofuranoside, methyl
48	11.1497	0.8553	Octane, 1-(ethenylthio)-
49	11.2150	0.4622	Benzaldehyde, 4-hydroxy-
50	11.3812	0.3539	3-Amino-4,5-dihydro-3H-[1,4']bipyridinyl-2,6-dione
54	11.7017	0.9917	Vanillin
55	11.9807	0.5968	2-Propenoic acid, 3-phenyl-
56	12.1766	1.7044	Heptanedioic acid
57	12.3546	1.6925	Cyclohexane, 1,3-dimethyl-, trans-
58	12.5861	2.2905	1-Decene
59	12.7107	0.4434	Oxazole, 2,4-dimethyl-
60	12.8116	0.2830	Apocynin
62	12.9659	1.0154	Propanoic acid, 3-hydroxy-
63	13.0609	0.4724	Propanoic acid, 3-hydroxy-
64	13.1440	0.5911	Benzoic acid, 4-hydroxy-
65	13.1855	0.9933	Benzoic acid, 4-hydroxy-
66	13.3280	1.3279	Octanedioic acid
67	13.3933	1.7386	2,2'-Heptamethylene-di-2-imidazoline
68	13.6188	0.5677	Pentadecanoic acid
69	13.7316	3.3262	Benzoic acid, 4-hydroxy-3-methoxy-
70	13.9156	0.4026	Nonanedioic acid, monomethyl ester
71	14.4557	2.1617	Azelaic acid (or nonanodioic acid)
72	14.6575	0.3131	3-(Ethyl-hydrazono)-butan-2-one
74	14.8118	0.3921	2-Propenamide, 3-phenyl-
75	14.8831	1.0172	N-(Trifluoroacetyl)-N,O,O',O''-tetrakis(trimethylsilyl)norepinephrine
77	15.4113	0.9514	2H-Pyran-2-one, tetrahydro-4-hydroxy-6-pentyl-
79	15.6665	0.8425	Benzaldehyde, 3,4,5-trimethoxy-
80	15.8565	0.3840	Trehalose
81	15.9930	0.4175	Thiazolo[5,4-d]pyrimidine, 5-methyl-
82	16.1829	0.3554	Cyclooctane, 1,2-dimethyl-

86	17.0494	0.2255	2-Propenoic acid, 3-(4-hydroxy-3-methoxyphenyl)-
88	17.5836	1.0127	Hexadecanoic acid, methyl ester
89	17.7261	0.2740	1-Tetradecene
91	17.9041	0.8591	n-Hexadecanoic acid
92	18.0644	1.3185	Scopoletin
94	18.4145	0.3436	Cycloheptadecanone
95	18.7588	0.2692	Benzonitrile, 4-(2-chlorobenzylidenamino)-
96	19.0793	1.4995	Acetic acid, 3,7,11,15-tetramethyl-hexadecyl ester
98	19.2752	0.3104	trans-3,4-Dimethoxy-2-ethoxy-.beta.-methylstyrene
100	19.4948	1.6066	Methyl stearate
102	19.7856	0.4921	Octadecanoic acid
104	19.9993	2.2008	Cyclopentadecane
108	20.7174	1.5143	Octadecanedioic acid

Table S2. Examples of antimicrobial activity reported in the literature for other natural products rich in glycerin, vanillic acid, and azabicyclo derivatives.

Phytochemical	Plant	Microorganism	Effectiveness	Ref.
Glycerin	*Cynodon dactylon*	*Streptococcus pyogenes*	IZ = 18 mm	[1]
		Staphylococcus aureus	IZ = 30 mm	
		Escherichia coli	IZ = 25 mm	
		Proteus mirabilis	IZ = 23 mm	
		Salmonella typhi	IZ = 11 mm	
	Salvadora persica	*S. aureus*	IZ = 6.635 mm	[2]
		Aspergillus terreus	IZ = 6.77 mm	
	Aphelandra squarrosa	*E. coli*	IZ = 14 mm	[3]
Vanillic acid	*Casuarina equisetifolia* leaves	*E. coli*	MIC = 25,000-100,000 $\mu g \cdot mL^{-1}$	[4]
		Klebsiella pneumoniae	MIC =25,000-50,000 $\mu g \cdot mL^{-1}$	
		P. aeruginosa	MIC = 50,000 $\mu g \cdot mL^{-1}$	
		Bacillus subtilis	MIC = 50,000-150,000 $\mu g \cdot mL^{-1}$	
		S. aureus	MIC = 25,000-100,000 $\mu g \cdot mL^{-1}$	
		*Micrococcu*s	MIC = 50,000-150,000 $\mu g \cdot mL^{-1}$	
	Onosma hispidum root bark, 500 $\mu g \cdot mL^{-1}$	*B. subtilis*	n.a.	[5]
		Corynebacterium diphtheriae	IZ = 20 mm	
		C. diphtheriticum	IZ = 19-20 mm	
		M. lysodiecticus	IZ = 20 mm	
		S. aureus	IZ = 19-20 mm	
		S. epidermidis	IZ = 20 mm	
		S. saprophyticus	IZ = 17-18 mm	
		Enterococcus faecalis	IZ = 20 mm	
		E. faecalis 2400	IZ = 18 mm	
		E. faecium	IZ = 18 mm	
		Streptococcus pneumoniae	IZ = 20 mm	
		S. pyogenes	IZ = 18-20 mm	
		E. coli WT	n.a.	
		E. coli BU40	n.a.	
		E. coli FPL5014	n.a.	
		K. pneumoniae	n.a.	
		P. mirabilis	n.a.	
		P. aeruginosa PAO286	n.a.	
		S. typhi	n.a.	
		S. paratyphi A	n.a.	
		S. paratyphi B	n.a.	
		Shigella dysenteriae	n.a.	
		S. sonneie	n.a.	
		S. flexneriae	n.a.	
	Ruta chalepensis stems	*S. aureus* ATCC 25923	IZ = 14.7-16.3 mm	[6]
		E. coli ATCC 35218	IZ = 13.3-17.3 mm	
		P. aeruginosa ATCC 27853	IZ = 7.7-17.7 mm	
	R. chalepensis leaves	*S. aureus* ATCC 25923	IZ = 12.3-15.3 mm	
		E. coli ATCC 35218	IZ = 14.3-16.3 mm	
		P. aeruginosa ATCC 27853	IZ = 9.7-16.7 mm	
	R. chalepensis flowers	*S. aureus* ATCC 25923	IZ = 15-16 mm	
		E. coli ATCC 35218	IZ = 13-15.7 mm	
		P. aeruginosa ATCC 27853	IZ = 15-16.3 mm	
	Pinus pinaster bark	*Acinetobacter baumannii*	MIC = 200,000 $\mu g \cdot mL^{-1}$	[7]
Azabicyclo derivates	*Azadirachta indica* + *Ocimum sanctum* leaves	*Aeromonas hydrophila*	MIC = 3200 $\mu g \cdot mL^{-1}$	[8]
		S. aureus	MIC = 3460 $\mu g \cdot mL^{-1}$	
		S. epidermidis	MIC = 4200 $\mu g \cdot mL^{-1}$	
		P. aeruginosa	MIC = 4820 $\mu g \cdot mL^{-1}$	
		Vibrio harveyi	MIC = 3750 $\mu g \cdot mL^{-1}$	

		V. vulnificus	MIC = 4460 µg·mL^{-1}	
	Melia dubia leaves, 50 µg·mL^{-1}	*E. coli*	IZ = 16 mm	[9]
		S. aureus	IZ = 15mm	
	Dioscorea hispida tuber, 50,000 µg·mL^{-1}	*S. aureus* ATCC 25923	IZ > 6-8 mm	[10]
		E.coli ATCC 25922	IZ > 6 mm	

IZ: inhibition zone; MIC: Minimum Inhibitory Concentration; n.a.: not activity.

Table S3. Inhibition values reported in the literature for *Q. suber* extracts against pathogenic microorganisms.

Collection site	Part of the plant	Solvent	Microorganisms	Effectiveness	Ref.
Jijel Algeria	Acorn	Acetone (70%)	*Candida albicans*	MIC = 105 $\mu g \cdot mL^{-1}$	[11]
			C. krusei	MIC = 100 $\mu g \cdot mL^{-1}$	
			C. guilliermondii	MIC = 80 $\mu g \cdot mL^{-1}$	
			Trichophyton verrucosum	MIC = 20 $\mu g \cdot mL^{-1}$	
			T. mentagrophytes	MIC = 20 $\mu g \cdot mL^{-1}$	
			Epidermophyton floccosum	MIC = 45 $\mu g \cdot mL^{-1}$	
			Aspergillus flavus	MIC = 60 $\mu g \cdot mL^{-1}$	
Bejaia, Algeria	Bark	Methanol:water (1:1), 3000 $\mu g \cdot mL^{-1}$	*Staphylococcus aureus ATCC 25923*	IZ = 12.1±0.5 mm	[12]
			Listeria innocua innocua CLIP 74915	n.a.	
			Escherichia coli ATCC 25922	n.a.	
			Pseudomonas aeruginosa ATCC 27853	IZ =10.07±0.1 mm	
Rabat, Morocco	Bark	Methanol	*C. albicans* L13 (IHEM 15835)	MIC = 25,000 $\mu g \cdot mL^{-1}$	[13]
			C. albicans L2 (IHEM 15824)	MIC = 25,000 $\mu g \cdot mL^{-1}$	
			C. albicans L5 (IHEM 15827)	MIC = 25,000 $\mu g \cdot mL^{-1}$	
			C. albicans L14 (IHEM 15836)	MIC = 12,500 $\mu g \cdot mL^{-1}$	
			C. albicans L12 (IHEM 15834)	MIC = 50,000 $\mu g \cdot mL^{-1}$	
n.s.	Bark	Methanol	*T. rubrum* M143	MIC = 50,000 $\mu g \cdot mL^{-1}$	[14]
			C. albicans L5	MIC = 12,500 $\mu g \cdot mL^{-1}$	
	Leaf		*T. rubrum* M143	MIC = 50,000 $\mu g \cdot mL^{-1}$	
			C. albicans L5	MIC = 50,000 $\mu g \cdot mL^{-1}$	
Tamil Nadu, India	Leaf	Methanol, 120 $\mu g \cdot mL^{-1}$	*Bacillus subtilis*	IZ = 12 mm	[15]
			E. coli	IZ = 13 mm	
			Streptococcus pneumoniae	IZ = 11 mm	
			S. aureus	IZ = 12 mm	
			A. niger	IZ = 14 mm	
			Penicillium sp	IZ = 12 mm	
			Fusarium oxysporum	IZ = 15 mm	
	Stem		*B. subtilis*	IZ = 12 mm	
			E. coli	IZ = 25 mm	
			S. pneumonia	IZ = 15 mm	
			S. aureus	IZ = 16 mm	
			Aspergillus niger	IZ = 14 mm	
			Penicillium sp	IZ = 14 mm	
			F. oxysporum	IZ = 20 mm	

IZ: inhibition zone; MIC: Minimum Inhibitory Concentration; n.a.: no activity at the highest concentration tested; n.s.: not specified

Table S4. Inhibitory values reported in the literature for bioactive natural substances against the pathogens under study.

Pathogen	Source/Solvent extraction	Natural Product	Inhibitory Value	Ref.
Monilinia fructigena	Aqueous ammonia extract (1:1)	*Quercus suber* bark extract	MIC = 1500 μg·mL^{-1}	This work
		COS–*Q. suber* bark	MIC = 1000 μg·mL^{-1}	
	Commercial EOs	*Mentha pulegium* leaves and flowers	EC_{50} = 18.87 μL·mL^{-1}	[16]
		Eucalyptus radiata flowers	EC_{50} = 15.34 μL·mL^{-1}	
		Lavandula angustifolia leaves and flowers	EC_{50} = 21.28 μL·mL^{-1}	
		Origanum compactum leaves and flowers	EC_{50} = 17.75 μL·mL^{-1}	
		Rosmarinus officinalis leaves and flowers	EC_{50} = 16.79 μL·mL^{-1}	
		Syzygium aromaticum flowers buds	EC_{50} = 10.09 μL·mL^{-1}	
		Thymus vulgaris leaves and flowers	EC_{50} = 12.52 μL·mL^{-1}	
		Citrus aurantium L. ssp. *amara* blossoms	EC_{50} = 10.36 μL·mL^{-1}	
		C. sinensis peel	EC_{50} = 11.19 μL·mL^{-1}	
	n-hexane	*Dittrichia viscosa* young shoots	MIC = 200 μg·mL^{-1}	[17]
		Ferula communis aerial part	MIC > 400 μg·mL^{-1}	
		F. communis root	MIC = 400 μg·mL^{-1}	
	Methanol or n-hexane	*Prunus laurocerasus* fruits	MIC > 1000 μg·mL^{-1}	[18]
		P. laurocerasus leaves	MIC > 1000 μg·mL	
		Cornus mas fruits	MIC > 1000 μg·mL^{-1}	
		C. mas leaves	MIC > 1000 μg·mL^{-1}	
		C. mas fruits seeds	MIC > 1000 μg·mL^{-1}	
		Morus nigra immature fruits	MIC > 1000 μg·mL^{-1}	
		M. nigra leaves	MIC > 1000 μg·mL^{-1}	
		M. alba immature fruits	MIC > 1000 μg·mL^{-1}	
		M. alba leaves	MIC > 1000 μg·mL^{-1}	
		Rosa canina fruits	MIC > 1000 μg·mL^{-1}	
	Water	*Punica granatum* peel	MIC = 50,000 μg·mL^{-1}	[19]
	Ethyl alcohol (96 %) (2000 μg·mL^{-1})	*Salix alba* bark	IR = 20%	[20]
		S. alba leaves	n.a.	
		Equisetum arvense	n.a	
		Artemisia absinthium aerial parts	IR = 20%	
		A. vulgaris aerial parts	IR = 30%	
M. laxa	Aqueous ammonia extract (1:1)	*Q. suber* bark extract	MIC = 1500 μg·mL^{-1}	This work
		COS–*Q. suber* bark	MIC = 750 μg·mL^{-1}	
	Commercial EOs	*M. pulegium* leaves and flowers	EC_{50} = 21.43 μL·mL^{-1}	[16]
		E. radiata flowers	EC_{50} = 20.80 μL·mL^{-1}	
		L. angustifolia leaves and flowers	EC_{50} = 21.23 μL·mL^{-1}	
		O. compactum leaves and flowers	EC_{50} = 20.20 μL·mL^{-1}	
		R. officinalis leaves and flowers	EC_{50} = 17.30 μL·mL^{-1}	
		S. aromaticum flowers buds	EC_{50} = 6.74 μL·mL^{-1}	
		T. vulgaris leaves and flowers	EC_{50} = 14.38 μL·mL^{-1}	

		C. aurantium subsp. *amara* blossoms	EC$_{50}$ = 10.96 μL·mL^{-1}	
		C. sinensis peel	EC$_{50}$ = 11.52 μL·mL^{-1}	
	n-hexane	*D. viscosa* young shoots	MIC = 200 μg·mL^{-1}	[17]
		F. communis aerial part	MIC > 400 μg·mL^{-1}	
		F. communis root	MIC = 400 μg·mL^{-1}	
	Water	*P. granatum* peel	MIC > 50,000 μg·mL^{-1}	[19]
	Ethyl alcohol (2000 μg·mL^{-1})	*S. alba* bark	IR = 20%	[20]
		S. alba leaves	IR = 0%	
		E. arvense	IR = 0%	
		A. absinthium aerial parts	IR = 22%	
		A. vulgaris aerial parts	IR = 22%	
N. parvum	Aqueous ammonia (1:1)	*Q. suber* bark extract	MIC > 1500 μg·mL^{-1}	This work
		COS–*Q. suber* bark	MIC = 750 μg·mL^{-1}	
	Water	*E. arvense*	MIC > 1500 μg·mL^{-1}	[21]
		Urtica dioica	MIC > 1500 μg·mL^{-1}	
	Methanol:water (1:1)	*Silybum marianum* capitula	MIC > 1500 μg·mL^{-1}	[22]
	Methanol:water (1:1)	*Rubia tinctorum* roots	MIC = 250 μg·mL^{-1}	[23]
P. cactorum	Aqueous ammonia extract (1:1)	*Q. suber* bark extract	MIC = 750 μg·mL^{-1}	This work
		COS–*Q. suber* bark	MIC = 375 μg·mL^{-1}	
	Aqueous ammonia extract (1:1)	*Uncaria tomentosa* bark	MIC = 187.5 μg·mL^{-1}	[24]
		COS–*U. tomentosa*	MIC = 39.05 μg·mL^{-1}	
	Commercial product	*Allium*-based extract	MIC = 100 μg·mL^{-1}	[25]
	Water extract	*Origanum heracleoticum* inflorescences	MIC > 500,000 μg·mL^{-1}	[26]
		S. officinalis leaves	MIC > 500,000 μg·mL^{-1}	
		R. officinalis leaves and flowers	MIC > 500,000 μg·mL^{-1}	
	Ethanol 96 %	*Pinus sylvestris* bark	MIC = 100 μg·mL^{-1}	[27]
		P. abies bark	MIC = 100 μg·mL^{-1}	
	Commercial EOs	*Eucalyptus. citriodora*	MIC > 28,000 μg·mL^{-1}	[28]
		Melaleuca quinquenervia	MIC > 28,000 μg·mL^{-1}	[29]
		Leptospermum pertersonii	MIC > 28,000 μg·mL^{-1}	
		Polylepis racemosa	MIC > 28,000 μg·mL^{-1}	
		Junierus oxycedrus	MIC > 28,000 μg·mL^{-1}	
		Cymbopogon nardus	MIC > 28,000 μg·mL^{-1}	
		Pelargonium graveolens	MIC = 28,000 μg·mL^{-1}	
		Cuminum cyminum	MIC > 28,000 μg·mL^{-1}	
		Myrristica fragrans	MIC > 28,000 μg·mL^{-1}	
		C. martini	MIC = 28,000 μg·mL^{-1}	
		M. pulegium	n.a.	
		M. spicata	n.a.	
		T. vulgaris	MIC = 14,000 μg·mL^{-1}	
E. amylovora	Aqueous ammonia (1:1)	*Q. suber* bark extract	MIC = 1000 μg·mL^{-1}	This work
		COS–*Q. suber* bark	MIC = 750 μg·mL^{-1}	
	Water	*Moringa oleifera* leaves	MIC > 2000 μg·mL^{-1}	[30]
	Hydroalcoholic extraction (ethanol:water, 70:30)		MIC = 1000 μg·mL^{-1}	
	Methanol extraction		MIC = 1000 μg·mL^{-1}	

	Water extract + Maltodextrins		MIC > 2000 μg·mL^{-1}	
	Hydroalcoholic extract with maltodextrins (50 % ethanol)		MIC = 1000 μg·mL^{-1}	
	Methanol/water (1:1, *v/v*)	*P. granatum* var. *nana* fruits	MIC = 1500 μg·mL^{-1}	[31]
	Methanol/water (1:1, *v/v*)	*Hibiscus syriacus* flowers	MIC = 750 μg·mL^{-1}	[32]
		H. syriacus leaves	MIC = 1000 μg·mL^{-1}	
	Methanol/water (1:1, *v/v*)	*Limonium binervosum* flowers	MIC = 1500 μg·mL^{-1}	[33]
		L. binervosum leaves	MIC = 1500 μg·mL^{-1}	
P. syringae pv. *syringae*	Aqueous ammonia (1:1)	*Q. suber* bark extract	MIC = 750 μg·mL^{-1}	
		COS–*Q. suber* bark	MIC = 375 μg·mL^{-1}	
	Ethanol, 10,000 μg·mL^{-1}	*Allium sativum* leaves	IZ = 19.5-23.4 mm	[34]
		Azadirachta indica leaves	IZ = 15.5-18.2 mm	
		A. cepa leaves	IZ = 13.2-14.6 mm	
		Ficus carica leaves	IZ = 10.2-12.4 mm	
		M. oleifera leaves	IZ = 17.25-24.4 mm	
		Psidium guajava leaves	IZ = 8.1-9.9 mm	
	Commercial EO	*T. vulgaris* leaves	MIC = 1400 μg·mL^{-1}	[35]
		O. vulgare leaves	MIC = 5800 μg·mL^{-1}	
	Commercial EO	*R. officinalis* aerial parts	MIC = 125 μg·mL^{-1}	[36]
		T. daenensis aerial parts	MIC = 3.92-15.68 μg·mL^{-1}	
		Foeniculum vulgare aerial parts	MIC = 62.72-125 μg·mL^{-1}	
		M. spicata aerial parts	MIC = 31.36-62.72 μg·mL^{-1}	
		M. piperita aerial parts	MIC = 62.72 μg·mL^{-1}	
		P. graveolens aerial parts	MIC = 62.72 μg·mL^{-1}	

IR: inhibition rate; IZ: inhibition zone diameter; MIC: Minimum Inhibitory Concentration; n.a.: not activity at the highest concentration tested; EC_{50}: effective concentration 50%.

References (reference numbers do not match those that appear in the main document)

1. Jatin R, R.; Priya R, S. Determination of bioactive components of *Cynodon dactylon* by GC-MS analysis & its in vitro antimicrobial activity. *International Journal of Pharmacy & Life Sciences* **2016**, *7*, 4880-4885.
2. Hameed, R.H.; Mohammed, G.J.; Hameed, I.H. Characterization of antimicrobial metabolites produced by *Salvadora persica* and analysis of its chemical compounds using GC-MS and FTIR. *Indian Journal of Public Health Research & Development* **2018**, *9*, 241, doi:10.5958/0976-5506.2018.00216.4.
3. Maria, K.K.; Joanna, G.K.; Katerina, A.D.; Leland, G.K. Chemical composition and antibacterial activity against *Escherichia coli* of extracts of a common household plant. *Journal of Medicinal Plants Research* **2021**, *15*, 56-63, doi:10.5897/jmpr2020.7031.
4. Nehad, M.G.; Abdulrahaman, S.H. Antimicrobial efficacy of *Casuarina equisetifolia* extracts against some pathogenic microorganisms. *Journal of Medicinal Plants Research* **2012**, *6*, 5819-5825.
5. Naz, S.; Ahmad, S.; Ajaz Rasool, S.; Asad Sayeed, S.; Siddiqi, R. Antibacterial activity directed isolation of compounds from *Onosma hispidum*. *Microbiol. Res.* **2006**, *161*, 43-48, doi:10.1016/j.micres.2005.05.001.
6. Ouerghemmi, I.; Bettaieb Rebey, I.; Rahali, F.Z.; Bourgou, S.; Pistelli, L.; Ksouri, R.; Marzouk, B.; Saidani Tounsi, M. Antioxidant and antimicrobial phenolic compounds from extracts of cultivated and wild-grown Tunisian *Ruta chalepensis*. *Journal of Food and Drug Analysis* **2017**, *25*, 350-359, doi:10.1016/j.jfda.2016.04.001.
7. Ćurković-Perica, M.; Hrenović, J.; Kugler, N.; Goić-Barišić, I.; Tkalec, M. Antibacterial activity of *Pinus pinaster* bark extract and its components against multidrug-resistant clinical isolates of *Acinetobacter baumannii*. *Croat. Chem. Acta* **2015**, *88*, 133-137, doi:10.5562/cca2548.
8. Harikrishnan, R.; Kim, M.-C.; Kim, J.-S.; Balasundaram, C.; Jawahar, S.; Heo, M.-S. Identification and antimicrobial activity of combined extract from *Azadirachta indica* and *Ocimum sanctum*. *Israeli Journal of Aquaculture - Bamidgeh* **2010**, *62*, 85-95.
9. Mudhafar, M.; Zainol, I.; Jaafar, C.; Alsailawi, H.; Majhool, A.A.; Alsaady, M. Phytochemical screening and characterization of *Melia dubia* leaves extract for antimicrobial activity against *Escherichia coli* and *Staphylococcus aureus*. *Indian Journal of Ecology* **2020**, *47*, 493-496.
10. Suryowati, T.; Sirait, R.H.; Siagian, F.E.; Nursyam, M. Bioactive compound impacting the metabolism and antibacterial activity of gadung tuber (*Dioscorea hispida* Dennst). *Journal of Physics: Conference Series* **2020**, *1665*, doi:10.1088/1742-6596/1665/1/012030.
11. Akroum, S. Antifungal activity of acetone extracts from *Punica granatum* L., *Quercus suber* L. and *Vicia faba* L. *Journal de Mycologie Médicale* **2017**, *27*, 83-89, doi:10.1016/j.mycmed.2016.10.004.
12. Touati, R.; Santos, S.A.O.; Rocha, S.M.; Belhamel, K.; Silvestre, A.J.D. The potential of cork from *Quercus suber* L. grown in Algeria as a source of bioactive lipophilic and phenolic compounds. *Industrial Crops and Products* **2015**, *76*, 936-945, doi:10.1016/j.indcrop.2015.07.074.
13. Lahlimi-Alami, Q.; Layachi, R.; Hassikou, R.; Benjelloun, J.; Amallah, L.; Guennoun, N.; Zaid, Y.; Bouzroud, S. Anticandidosic activity and acute toxicity of *Quercus suber* L. bark extracts. *Journal of Medicinal and Chemical Sciences* **2022**, *5*, 769-778, doi:10.26655/jmchemsci.2022.5.11.
14. Hassikou, R.; Oulladi, H.; Arahou, M. Activité antimycosique des extraits du chêne-liège *Quercus suber* sur *Trichophyton rubrum* et *Candida albicans*. *Phytothérapie* **2014**, *12*, 206-212, doi:10.1007/s10298-014-0874-2.
15. Subhashini, S.; Begum, S.M.; Rajesh, G. Antimicrobial characterisation combining spectrophotometric analysis of different oak species. *Int. J. Herb. Med* **2016**, *4*, 32-35.
16. El Khetabi, A.; Ezrari, S.; El Ghadraoui, L.; Tahiri, A.; Ait Haddou, L.; Belabess, Z.; Merah, O.; Lahlali, R. In vitro and in vivo antifungal activities of nine commercial essential oils against brown rot in apples. *Horticulturae* **2021**, *7*, 545, doi:10.3390/horticulturae7120545.
17. Mamoci, E.; Cavoski, I.; Simeone, V.; Mondelli, D.; Al-Bitar, L.; Caboni, P. Chemical composition and in vitro activity of plant extracts from *Ferula communis* and *Dittrichia viscosa* against postharvest fungi. *Molecules* **2011**, *16*, 2609-2625, doi:10.3390/molecules16032609.
18. Onaran, A.; Yanar, Y. *In vivo* and *in vitro* antifungal activities of five plant extracts against various plant pathogens. *Egyptian Journal of Biological Pest Control* **2016**, *26*, 405-411.
19. El Khetabi, A.; Lahlali, R.; Askarne, L.; Ezrari, S.; El Ghadaroui, L.; Tahiri, A.; Hrustić, J.; Amiri, S. Efficacy assessment of pomegranate peel aqueous extract for brown rot (*Monilinia* spp.) disease control. *Physiological and Molecular Plant Pathology* **2020**, *110*, 101482, doi:10.1016/j.pmpp.2020.101482.
20. Andreu, V.; Levert, A.; Amiot, A.; Cousin, A.; Aveline, N.; Bertrand, C. Chemical composition and antifungal activity of plant extracts traditionally used in organic and biodynamic farming.

Environmental Science and Pollution Research **2018**, *25*, 29971-29982, doi:10.1007/s11356-018-1320-z.

21. Langa-Lomba, N.; Buzón-Durán, L.; Martín-Ramos, P.; Casanova-Gascón, J.; Martín-Gil, J.; Sánchez-Hernández, E.; González-García, V. Assessment of conjugate complexes of chitosan and *Urtica dioica* or *Equisetum arvense* extracts for the control of grapevine trunk pathogens. *Agronomy* **2021**, *11*, 976, doi:10.3390/agronomy11050976.
22. Langa-Lomba, N.; Buzón-Durán, L.; Sánchez-Hernández, E.; Martín-Ramos, P.; Casanova-Gascón, J.; Martín-Gil, J.; González-García, V. Antifungal activity against Botryosphaeriaceae fungi of the hydro-methanolic extract of *Silybum marianum* capitula conjugated with stevioside. *Plants* **2021**, *10*, 1363, doi:10.3390/plants10071363.
23. Langa-Lomba, N.; Sánchez-Hernández, E.; Buzón-Durán, L.; González-García, V.; Casanova-Gascón, J.; Martín-Gil, J.; Martín-Ramos, P. Activity of anthracenediones and flavoring phenols in hydromethanolic extracts of *Rubia tinctorum* against grapevine phytopathogenic fungi. *Plants* **2021**, *10*, 1527, doi:10.3390/plants10081527.
24. Sánchez-Hernández, E.; Martín-Ramos, P.; Martín-Gil, J.; Santiago-Aliste, A.; Hernández-Navarro, S.; Oliveira, R.; González-García, V. Bark extract of *Uncaria tomentosa* L. for the control of strawberry phytopathogens. *Horticulturae* **2022**, *8*, 672, doi:10.3390/horticulturae8080672.
25. Oladejo, O.; Imani, J. Inhibitory effect of CUSTOS, a formulated allium-based extract, on the growth of some selected plant pathogens. *International Journal of Plant Biology* **2022**, *13*, 44-54, doi:10.3390/ijpb13020006.
26. Salamone, A.; Zizzo, G.V.; Scarito, G. The antimicrobial activity of water extracts from Labiatae. *Acta Horticulturae* **2006**, 10.17660/ActaHortic.2006.723.67, 465-470, doi:10.17660/ActaHortic.2006.723.67.
27. Minova, S.; Sešķēna, R.; Voitkāne, S.; Metla, Z.; Daugavietis, M.; Jankevica, L. Impact of pine (*Pinus sylvestris* L.) and spruce (*Picea abies* (L.) Karst.) bark extracts on important strawberry pathogens. *Proceedings of the Latvian Academy of Sciences. Section B. Natural, Exact, and Applied Sciences.* **2015**, *69*, 62-67, doi:10.1515/prolas-2015-0008.
28. Lee, Y.-S.; Kim, J.; Shin, S.-C.; Lee, S.-G.; Park, I.-K. Antifungal activity of Myrtaceae essential oils and their components against three phytopathogenic fungi. *Flavour and Fragrance Journal* **2008**, *23*, 23-28, doi:10.1002/ffj.1850.
29. Kim, J.; Lee, Y.S.; Lee, S.G.; Shin, S.C.; Park, I.K. Fumigant antifungal activity of plant essential oils and components from West Indian bay (*Pimenta racemosa*) and thyme (*Thymus vulgaris*) oils against two phytopathogenic fungi. *Flavour and Fragrance Journal* **2008**, *23*, 272-277, doi:10.1002/ffj.1882.
30. Fontana, R.; Macchi, G.; Caproni, A.; Sicurella, M.; Buratto, M.; Salvatori, F.; Pappadà, M.; Manfredini, S.; Baldisserotto, A.; Marconi, P. Control of *Erwinia amylovora* growth by *Moringa oleifera* leaf extracts: *in vitro* and *in planta* effects. *Plants* **2022**, *11*, doi:10.3390/plants11070957.
31. Sánchez-Hernández, E.; Buzón-Durán, L.; Cuchí-Oterino, J.A.; Martín-Gil, J.; Lorenzo-Vidal, B.; Martín-Ramos, P. Dwarf pomegranate (*Punica granatum* L. var. *nana*): Source of 5-HMF and bioactive compounds with applications in the protection of woody crops. *Plants* **2022**, *11*, 550, doi:10.3390/plants11040550.
32. Sánchez-Hernández, E.; Buzón-Durán, L.; Lorenzo-Vidal, B.; Martín-Gil, J.; Martín-Ramos, P. Physicochemical characterization and antimicrobial activity against *Erwinia amylovora, Erwinia vitivora*, and *Diplodia seriata* of a light purple *Hibiscus syriacus* L. cultivar. *Plants* **2021**, *10*, 1876, doi:10.3390/plants10091876.
33. Sánchez-Hernández, E.; Buzón-Durán, L.; Langa-Lomba, N.; Casanova-Gascón, J.; Lorenzo-Vidal, B.; Martín-Gil, J.; Martín-Ramos, P. Characterization and antimicrobial activity of a halophyte from the Asturian coast (Spain): *Limonium binervosum* (G.E.Sm.) C.E.Salmon. *Plants* **2021**, *10*, 1852, doi:10.3390/plants10091852.
34. Islam, M.S.; Sultana, R.; Hasan, M.A.; Alam, M.S.; Sikdar, B.; Kamaruzzaman, M.; Islam, M.A. Characterization and biocontrol measures of *Pseudomonas syringae* pv. *syringae* associated with citrus blast disease. *Vegetos* **2020**, *33*, 555-569, doi:10.1007/s42535-020-00138-1.
35. Carezzano, M.E.; Sotelo, J.P.; Primo, E.; Reinoso, E.B.; Paletti Rovey, M.F.; Demo, M.S.; Giordano, W.F.; Oliva, M.d.l.M.; Flemetakis, E. Inhibitory effect of *Thymus vulgaris* and *Origanum vulgare* essential oils on virulence factors of phytopathogenic *Pseudomonas syringae* strains. *Plant Biology* **2017**, *19*, 599-607, doi:10.1111/plb.12572.
36. Shabani, B.; Rezaei, R.; Charehgani, H.; Salehi, A. Study on antibacterial effect of essential oils of six plant species against *Pseudomonas syringae* pv. *syringae* Van Hall 1902 and *Pseudomonas fluorescens* Migula 1894. *Journal of Plant Pathology* **2019**, *101*, 671-675, doi:10.1007/s42161-019-00266-x.
